# Global, regional, and national burden of incidence, prevalence, and years lived with disability for facial fractures from 1990 to 2019: a systematic analysis for the Global Burden of Disease study 2019

**DOI:** 10.1186/s12903-024-04206-9

**Published:** 2024-04-10

**Authors:** Yi Yi, Xiao He, Yiping Wu, Dawei Wang

**Affiliations:** 1 Department of Plastic and Cosmetic Surgery, Tongji Hospital, Tongji Medical College, Huazhong University of Science and Technology, 1095 Jiefang Avenue, Wuhan, Hubei China; 2 Department of Dermatology, Peking University Third Hospital, Beijing, China

**Keywords:** Facial fractures, Global burden of disease, Incidence, Prevalence, YLDs

## Abstract

**Background:**

Facial fractures are common injuries causing cosmetic, functional, and psychological damage. The purpose of this study was to assess the incidence, prevalence, and years lived with disability (YLDs) of facial fractures from 1990 to 2019 using the Global Burden of Disease (GBD).

**Methods:**

Detailed data for the disease burden of facial fractures were obtained from online available public data (Global Health Data Exchange) derived from the GBD study. The incidence, prevalence, and YLDs of facial fractures from 1990 to 2019 were analyzed by country, region, age, gender, sociodemographic index (SDI), and cause. The age-standardized incidence rate (ASIR), age-standardized prevalence rate (ASPR), age-standardized YLDs rate (ASYR), and estimated annual percentage change (EAPC) were calculated to evaluate the disease burden and quantify the trends over time. The main causes of facial fractures in different years and ages were assessed.

**Results:**

Globally, there were 8.9 million incident cases, 1.5 million cases prevalent cases, and 98.1 thousand years YLDs in 2019. Compared with 1990, the number of incident cases, prevalent cases, and YLDs increased, while ASIR (EAPC, − 0.47; 95% uncertainty interval [UI], − 0.57 to − 0.37), ASPR (EAPC, − 0.39; 95% UI, − 0.46 to − 0.31), ASYR (EAPC, − 0.39; 95% UI, − 0.47 to − 0.32) showed a downward trend. The high SDI region held the highest ASIR, ASPR, and ASYR both in 1990 and 2019, such as New Zealand, Slovenia, and Australia. The burden was higher in men than in women from 1990 to 2019, while the ASRs in women exceeded that of men in the elderly. The ASIR peaked in the young adult group, however, the ASPR and ASYR increased with age. Falls and road injuries were the leading causes of facial fractures.

**Conclusions:**

Facial fractures continue to cause a heavy burden on public health worldwide. More targeted strategies need to be established to control the burden of facial fractures.

**Supplementary Information:**

The online version contains supplementary material available at 10.1186/s12903-024-04206-9.

## Introduction

Facial fractures occur at various sites, including the maxilla, mandible, zygomatic bone, temporal bone, nasal bone, and frontal bone [1]. As a common emergency, facial fractures can lead to function loss of vision, hearing, chewing, facial expression, and even death [2–4]. Besides, facial fractures seriously affect facial aesthetics, resulting in facial asymmetry, facial deformities, or psychosocial disorders [5, 6]. Thus, the goal of facial fracture treatment is to restore functional and aesthetic integrity. Open reduction and internal fixation (ORIF) is a surgical procedure that restores the dislocated fragments to their anatomical position through stable fixation [7, 8]. Titanium or absorbable plates are routinely used for stable fixation to recover early function and aesthetics. Treatment of facial fractures requires the involvement of plastic surgeons or oral-maxillofacial specialists, however, there is no timely access to effective medical care in poor areas.

The impact of facial fractures on public health is highlighted. In the United States, more than 400,000 emergency room visits for facial fractures occur annually at a cost of over $ 1 million [9]. The leading causes of facial fractures in the United States were reported as assault, falls, and motor vehicle collisions [9, 10]. Multiple studies have shown that facial fractures tend to occur in the male population aged 20–30 years [11–13]. Differently, an epidemiology study demonstrated that women were more prone to facial fractures than men among older adults, with falls being the most common cause [14]. Epidemiology and pattern of facial fractures vary across populations due to socioeconomic, cultural, and war factors [9, 11, 15] Nevertheless, epidemiological and therapeutic data on facial fractures remain limited worldwide, especially in developing or low-income countries.

The global epidemiology of facial fractures is essential for better injury prevention and improved resource allocation. Comprehensive assessments are required to identify the trends, causes, and risk factors of facial fractures, which can help to develop prevention strategies and health policies. However, there is a lack of up-to-date studies assessing the global burden of facial fractures in all countries and regions [16]. The Global Burden of Diseases (GBD) Study 2019 provides estimates of diseases and injuries burden (including facial fractures) in 204 countries and territories from 1990 to 2019 [17]. In this study, we used the data from GBD 2019 to analyze the incidence, prevalence and years lived with disability (YLD) of facial fractures by countries, regions, gender, age, sociodemographic index (SDI), and cause. This study provides the latest and comprehensive understanding of the burden of facial fractures to facilitate injury management and policy making.

## Methods

Data sources for the disease burden of facial fractures were collected from the Global Health Data Exchange (GHDx) online data source query tool (https://vizhub.healthdata.org/gbd-results/). The data on the incidence, prevalence, and YLDs of facial fractures from 1990 to 2019 in 21 regions and 204 countries/territories were extracted (last date: 6 August 2023). Both crude estimates and age-standardized rates (ASR) were collected and summarized regarding incidence, prevalence, and YLDs attributable to facial fractures [18, 19]. The global trend in facial fractures was analyzed in line with both genders and the following age groups: <5, 5–10, 10–15, 15–19, 20–24, 25–29, 30–34, 35–39, 40–44, 45–49, 50–54, 55–59, 60–64, 65–69, 70–74, 75–79, 80–84, 85–89, 90–94, and > 95 years old.

The age-standardized incidence rate (ASIR), age-standardized prevalence rate (ASPR), and age-standardized YLDs rate (ASYR) were calculated to evaluate the global burden of facial fractures. The ASIR or ASPR referred to the number of cases per 100,000 persons with adjusted for population age differences, while the ASYR represented the life years with disability per 100,000 people. The GBD website provided the sociodemographic index (SDI) of each country, which was a composite indicator combining per capita income, fertility rate, and education level. The associations between the SDI and ASR were calculated to explore the influencing factors of the ASR of facial fractures. Besides, the main causes of facial fractures in different years and ages were analyzed.

The estimated annual percentage change (EAPC) was calculated to quantify the trends of ASR over time with the raw data in this study [20]. The ASR was considered to show an increasing trend if the EAPC and the lower limit of 95% uncertainty interval (UI) were positive. Correspondingly, the ASR posed a decreasing trend when the EAPC and the upper limit of 95% UI were negative. EAPCs were calculated based on the linear regression model and natural logarithm fitting data. The formula is y = a + bx + e, where x is the calendar year and y refers to ln (ASR). EAPC was calculated as 100*(exp[b]-1), as well as its 95% UI from the linear regression model.

All statistical analyses were performed on the R software 4.2.1 (R Foundation for Statistical Computing, Vienna, Austria). *P* < 0.05 was considered to be statistically significant.

## Results

### Incidence of facial fractures

The global incident cases of facial fractures increased from 8.9 million cases (95% UI: 7.1 million to 11.4 million cases) in 1990 to 10.7 million cases (95% UI: 8.5 million to 13.5 million cases) in 2019, corresponding to an increase of 19% (95% UI: 10–26%). However, the global ASIR of facial fractures decreased from 161.5 (95% UI: 128.8 to 204.8) per 100,000 in 1990 to 138.8 (95% UI: 110.6 to 174.8) per 100,000 in 2019 with an EAPC of − 0.47 (95% UI: −0.57 to − 0.37).

**Fig. 1:**
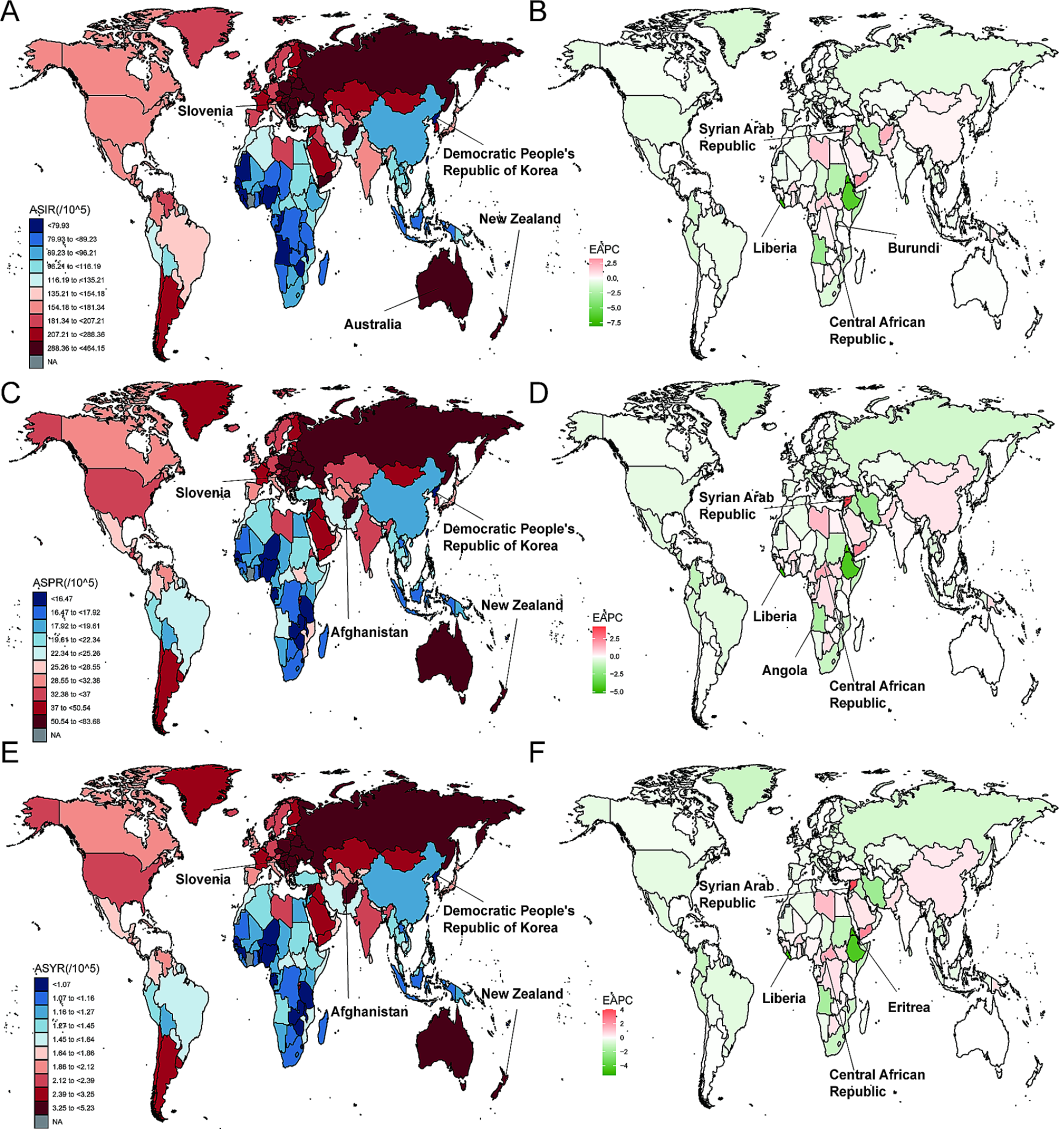
ASR and EAPC of facial fractures in 204 countries and territories. The ASIR (**A**), ASPR (**C**), and ASYR (**E**) in 2019. The EAPC in ASIR (**B**), ASPR (**D**), and ASYR (**F**) from 1990 to 2019. ASR, age-standardized rate; EAPC, estimated annual percentage changes; YLDs, years lived with disability; ASIR, age-standardized incidence rate; ASPR, age-standardized prevalence rate; ASYR, age-standardized YLDs rate

In 2019, the ASIR across the 204 countries was highest in New Zealand (464.2 per 100,000, 95% UI: 350.1 to 620.6 per 100,000), followed by Slovenia (401.1 per 100,000, 95% UI: 303.1 to 538.4 per 100,000), Australia (400.2 per 100,000, 95% UI: 295.7 to 539.4 per 100,000), while lowest in Democratic People’s Republic of Korea (59.2 per 100,000, 95% UI: 47.2 to 73.8 per 100,000) (Fig. 1A, Supplementary Table S1). The EAPC in the incidence was highest in the Syrian Arab Republic (8.19, 95% UI: 5.25 to 11.22) and Central African Republic (3.80, 95% UI: 2.09 to 5.54), while lowest in Liberia (-6.33, 95% UI: -8.85 to -3.74) and Burundi (-5.83, 95% UI: -9.48 to -2.04) (Fig. 1B).

**Fig. 2:**
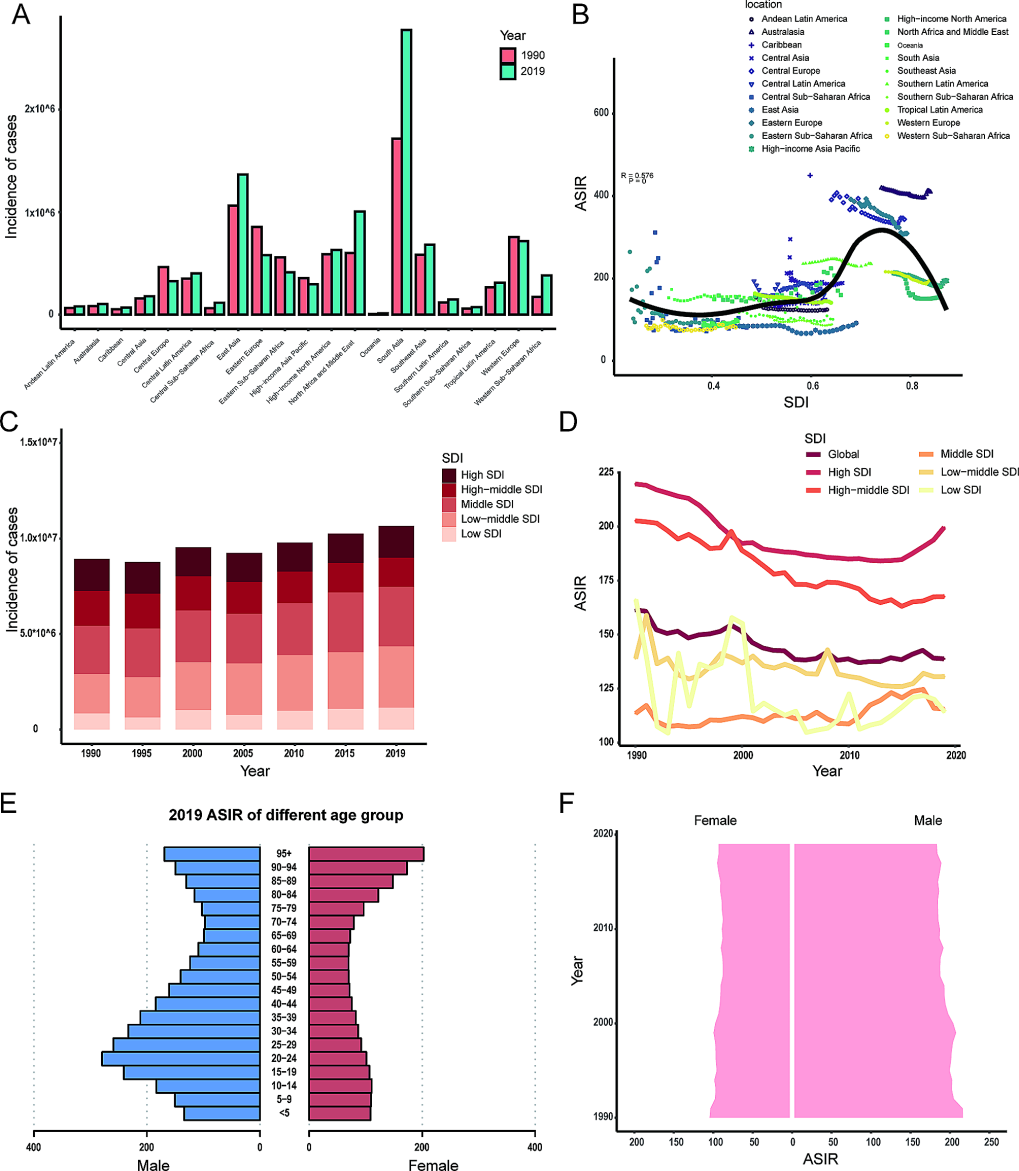
The incidence of facial fractures. (**A**) The number of incident cases between 1990 and 2019 in 21 regions; (**B**) the association between ASIR and SDI in 21 regions; (**C**) changes in incident cases in different SDI regions from 1990 to 2019; (**D**) trends in ASIR in different SDI regions from 1990 to 2019; (**E**) the ASIR of different age group in 2019; (**F**) the ASIR in both sexes from 1990 to 2019. ASIR, age-standardized incidence rate; SDI, sociodemographic index

In terms of geographic regions, the number of incident cases has increased in most regions, except Eastern Europe (-32%, 95% UI: -34% to -29%), Central Europe (-29%, 95% UI: -32% to -26%), Eastern Sub-Saharan Africa (-26%, 95% UI: -63% to -34%), High-income Asia Pacific (-17%, 95% UI: -21% to -14%), and Western Europe (-17%, 95% UI: -21% to -14%) (Supplementary Table S2, Fig. 2A). In 2019, the ASIR was the highest in Australasia (410.1 per 100,000, 95% UI: 304.4 to 552.8 per 100,000) and the lowest in Central Sub-Saharan Africa (80.3 per 100,000, 95% UI: 63.7 to 100.3 per 100,000). The ASIR showed a decreasing trend in most regions from 1990 to 2019, except North Africa and Middle East (EAPC, 1.04, 95% UI: 0.61 to 1.47) and Caribbean (EAPC, 0.54, 95% UI: -0.35 to 1.43) (Supplementary Table S2).

There was a significant positive correlation between ASIR and SDI (ρ = 0.58, *p* < 0.001) (Fig. 2B). The number of incident cases showed an increasing trend from 1990 to 2019 in all SDI regions except high-middle SDI region (-6%, 95% UI: -10% to -1%) (Fig. 2C). The middle SDI region had the largest incident cases in 1990 (2.1 million, 95% UI: 1.6 million to 2.6 million) and 2019 (2.7 million, 95% UI: 2.2 million to 3.4 million). The high SDI region held the highest ASIR both in 1990 (219.8, 95% UI: 167.8 to 287.3 per 100,000) and in 2019 (199.6, 95% UI: 150.6 to 260.9 per 100,000) (Fig. 2D). The ASIR decreased in all SDI regions from 1990 to 2019 except middle SDI region (EAPC, 0.31, 95% UI: 0.17 to 0.45). In 2019, the ASIR of facial fractures varied among the different age groups, reaching a peak in the 20–24 years age group in males and 10–14 years age group in females (Fig. 2E). Further, there was a gradual increase in incidence rate with age higher than 70 years old. The ASIR was higher in males than in females from 1990 to 2019 (Fig. 2F). Specifically, there were 216.6 per 100,000 males versus 104.7 per 100,000 females in 1990, while 183.4 per 100,000 males versus 93.0 per 100,000 females in 2019.

### Prevalence of facial fractures

The global prevalent cases of facial fractures increased from 1.5 million cases (95% UI: 1.2 million to 1.8 million cases) in 1990 to 2.1 million cases (95% UI: 1.8 million to 2.5 million cases) in 2019, corresponding to an increase of 42% (95% UI: 34–48%). However, the global ASPR of facial fractures decreased from 30.1 (95% UI: 25.4 to 36.0) per 100,000 in 1990 to 27.1 (95% UI: 23.0 to 31.9) per 100,000 in 2019 with an EAPC of − 0.39 (95% UI: −0.46 to − 0.31).

In 2019, the ASPR across the 204 countries was highest in Afghanistan (83.7 per 100,000, 95% UI: 36.3 to 198.1 per 100,000), followed by New Zealand, Slovenia, while lowest in Democratic People’s Republic of Korea (11.4 per 100,000, 95% UI: 9.7 to 13.3 per 100,000) (Fig. 1C, Supplementary Table S1). The EAPC in the prevalence was highest in the Syrian Arab Republic (7.49, 95% UI: 5.07 to 9.96) and Central African Republic (3.27, 95% UI: 2.02 to 4.54), while lowest in Liberia (-3.59, 95% UI: -5.21 to -1.94) and Angola (-2.41, 95% UI: -3.39 to -1.43) (Fig. 1D).

**Fig. 3:**
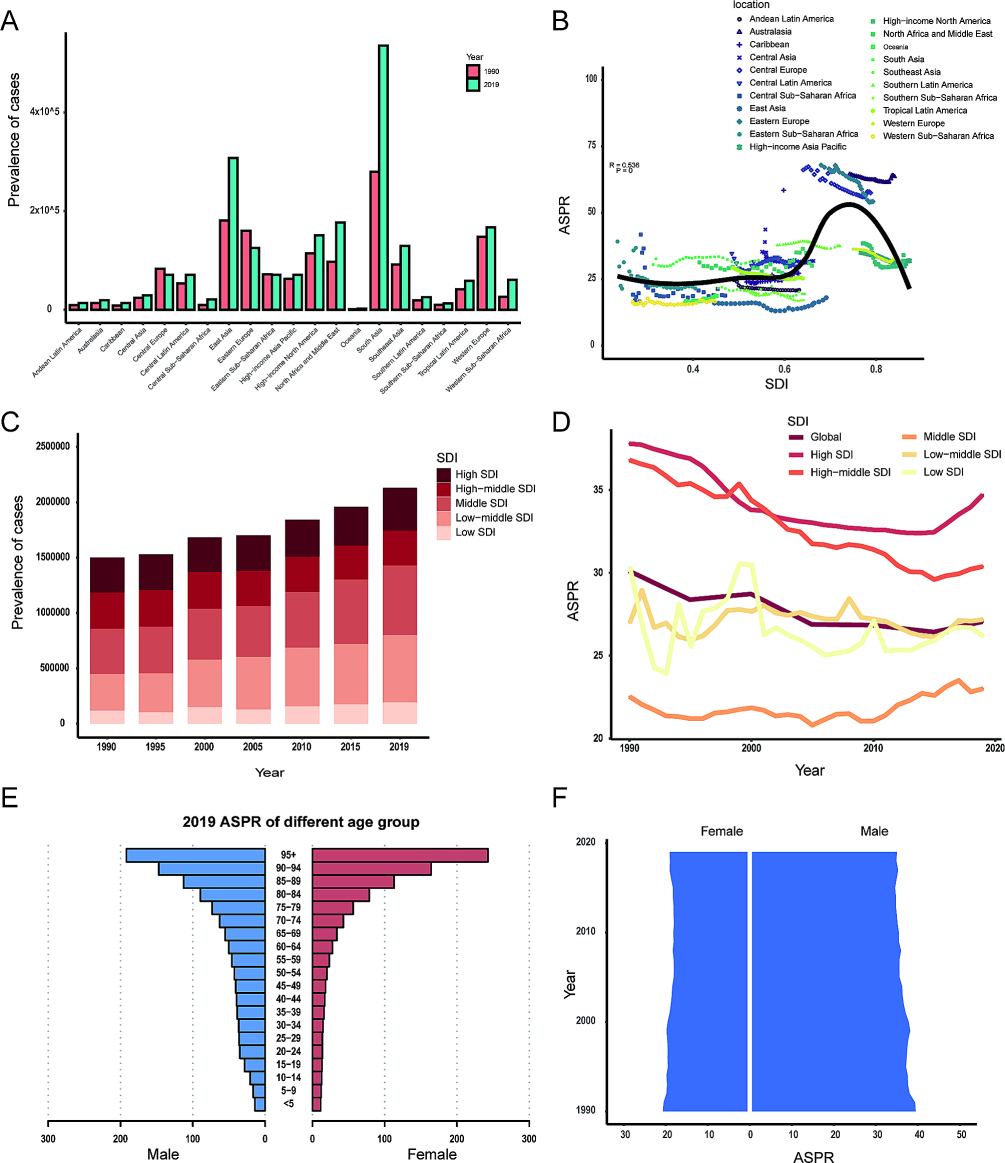
The prevalence of facial fractures. (**A**) The number of prevalent cases between 1990 and 2019 in 21 regions; (**B**) the association between ASPR and SDI in 21 regions; (**C**) changes in prevalent cases in different SDI regions from 1990 to 2019; (**D**) trends in ASPR in different SDI regions from 1990 to 2019; (**E**) the ASPR of different age group in 2019; (**F**) the ASPR in both sexes from 1990 to 2019. ASPR, age-standardized prevalence rate; SDI, sociodemographic index

In terms of geographic regions, the number of prevalent cases has increased in most regions, except Central Europe (-63%, 95% UI: -73% to -53%), Eastern Europe (-22%, 95% UI: -24% to -19%), and Eastern Sub-Saharan Africa (-1%, 95% UI: -46–55%) (Supplementary Table S2, Fig. 3A). In 2019, the ASPR was the highest in Australasia (63.6 per 100,000, 95% UI: 51.0 to 82.0 per 100,000) and the lowest in Western Sub-Saharan Africa (16.7 per 100,000, 95% UI: 14.1 to 19.7 per 100,000). The ASPR showed decreasing trend in most regions from 1990 to 2019, except Caribbean (EAPC, 0.75, 95% UI: 0.12 to 1.39), North Africa and Middle East (EAPC, 0.42, 95% UI: 0.14 to 0.71), Oceania (EAPC, 0.33, 95% UI: 0.06 to 0.59), South Asia (EAPC, 0.16, 95% UI: 0 to 0.31), Western Sub-Saharan Africa (EAPC, 0.09, 95% UI: -0.06 to 0.25), and Central Latin America (EAPC, 0.02, 95% UI: -0.21 to 0.25) (Supplementary Table S2).

There was a significant positive correlation between ASPR and SDI (ρ = 0.54, *p* < 0.001) (Fig. 3B). The number of prevalent cases showed an increasing trend from 1990 to 2019 in all SDI regions (Fig. 3C). The high SDI region held the highest ASPR both in 1990 (37.8, 95% UI: 31.2 to 46.5 per 100,000) and in 2019 (34.7, 95% UI: 28.6 to 42.8 per 100,000) (Fig. 3D). The ASPR decreased in all SDI regions from 1990 to 2019 except middle SDI region (EAPC, 0.18, 95% UI: 0.06 to 0.30). In 2019, the ASPR of facial fractures increased with age (Fig. 3E). The ASPR was higher in males than in females from 1990 to 2019 (Fig. 3F). Specifically, there were 39.5 per 100,000 males versus 20.7 per 100,000 females in 1990, while 35.0 per 100,000 males versus 19.1 per 100,000 females in 2019.

### YLDs of facial fractures

The global YLDs of facial fractures increased from 98.1 thousand years (95% UI: 58.5 thousand to 145.8 thousand years) in 1990 to 137.6 thousand years (95% UI: 84.3 thousand to 201.4 thousand years) in 2019, corresponding to an increase of 40% (95% UI: 32–47%). However, the global ASYR of facial fractures decreased from 2.0 (95% UI: 1.2 to 2.9) per 100,000 in 1990 to 1.8 (95% UI: 1.1 to 2.6) per 100,000 in 2019 with an EAPC of − 0.39 (95% UI: −0.47 to − 0.32).

In 2019, the ASYR across the 204 countries was highest in Afghanistan (5.2 per 100,000, 95% UI: 2.1 to 11.5 per 100,000), followed by New Zealand, Slovenia, while lowest in Democratic People’s Republic of Korea (0.7 per 100,000, 95% UI: 0.5 to 1.1 per 100,000) (Fig. 1E, Supplementary Table S1). The EAPC in the YLDs was highest in the Syrian Arab Republic (7.48, 95% UI: 5.05 to 9.96) and Central African Republic (3.29, 95% UI: 2.02 to 4.58), while lowest in Liberia (-3.74, 95% UI: -5.41 to -2.04) and Eritrea (-3.19, 95% UI: -4.42 to -1.93) (Fig. 1F).

**Fig. 4:**
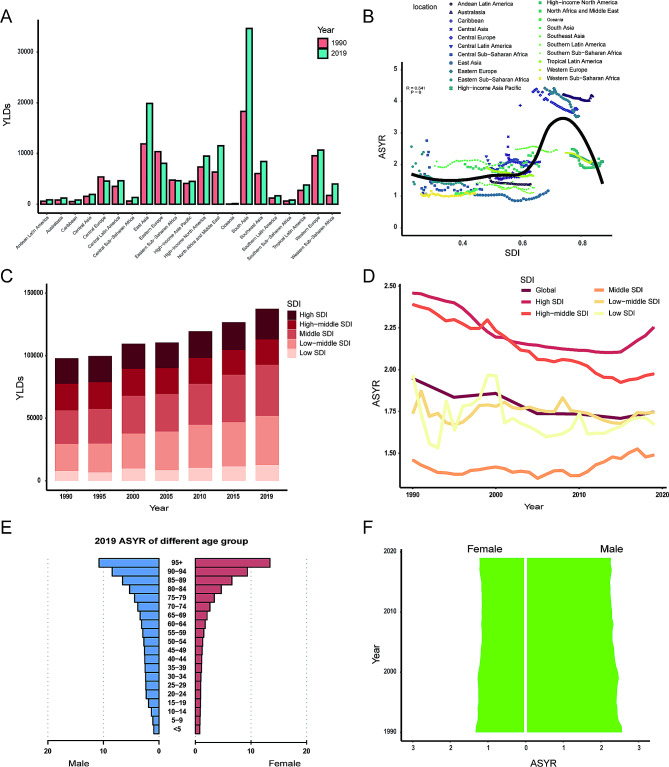
The YLDs of facial fractures. (**A**) The number of YLDs between 1990 and 2019 in 21 regions; (**B**) the association between ASYRand SDI in 21 regions; (**C**) changes in YLDs in different SDI regions from 1990 to 2019; (**D**) trends in ASYRin different SDI regions from 1990 to 2019; (**E**) the ASYRof different age group in 2019; (**F**) the ASYRin both sexes from 1990 to 2019. ASYR, age-standardized YLDs rate; SDI, sociodemographic index; YLDs, years lived with disability

In terms of geographic regions, the number of YLDs has increased in most regions, except Eastern Sub-Saharan Africa (-2%, 95% UI: -46% to -55%), Central Europe (-16%, 95% UI: -20% to -11%), and Eastern Europe (-22%, 95% UI: -25–19%) (Supplementary Table S2, Fig. 4A). In 2019, the ASYR was the highest in Australasia (4.2 per 100,000, 95% UI: 2.4 to 6.3 per 100,000) and the lowest in Western Sub-Saharan Africa (1.1 per 100,000, 95% UI: 0.7 to 1.6 per 100,000). The ASYR showed a decreasing trend in most regions from 1990 to 2019, except Caribbean (EAPC, 0.72, 95% UI: 0.08 to 1.38), North Africa, and Middle East (EAPC, 0.46, 95% UI: 0.17 to 0.76), Oceania (EAPC, 0.31, 95% UI: 0.03 to 0.58), South Asia (EAPC, 0.15, 95% UI: 0.01 to 0.30), Western Sub-Saharan Africa (EAPC, 0.09, 95% UI: -0.07 to 0.25), and Central Latin America (EAPC, 0.03, 95% UI: -0.20 to 0.26) (Supplementary Table S2).

There was a significant positive correlation between ASYR and SDI (ρ = 0.54, *p* < 0.001) (Fig. 4B). The number of YLDs showed an increasing trend from 1990 to 2019 in all SDI regions (Fig. 4C). The high SDI region held the highest ASYR both in 1990 (2.5, 95% UI: 1.5 to 3.7 per 100,000) and in 2019 (2.3, 95% UI: 1.3 to 3.4 per 100,000) (Fig. 4D). The ASYR decreased in all SDI regions from 1990 to 2019 except middle SDI region (EAPC, 0.18, 95% UI: 0.07 to 0.30). In 2019, the ASYR of facial fractures increased with age (Fig. 4E). The ASYR was higher in males than in females from 1990 to 2019 (Fig. 4F). Specifically, there were 2.6 per 100,000 males versus 1.3 per 100,000 females in 1990, while 2.3 per 100,000 males versus 1.2 per 100,000 females in 2019.

### Causes of facial fractures

**Fig. 5:**
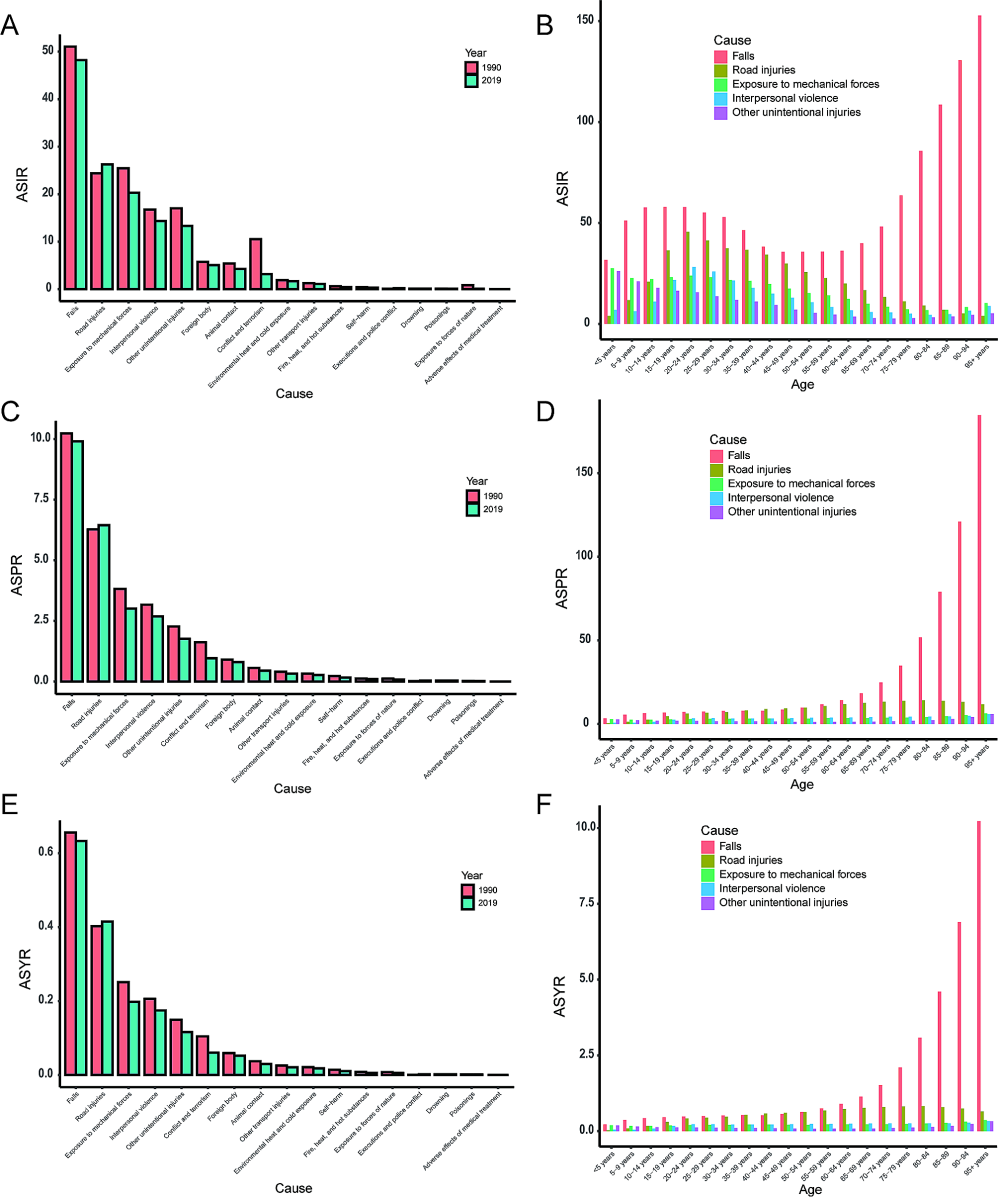
The causes of facial fractures. The cause composition of the ASIR (**A**), ASPR (**C**), and ASYR (**E**) of facial fractures between 1990 and 2019. The top five causes for the incidence (**B**), prevalence (**D**), and YLDs (**F**) of facial fractures in different age groups in 2019. YLDs, years lived with disability; ASIR, age-standardized incidence rate; ASPR, age-standardized prevalence rate; ASYR, age-standardized YLDs rate

The five leading causes for the incidence, prevalence, and YLDs of facial fractures included falls, road injuries, exposure to mechanical forces, interpersonal violence, and other unintentional injuries (Fig. 5). Falls were the leading cause of facial fractures, with an ASIR of 48.2 (95% UI: 27.3 to 77.4) per 100,000 in 2019, an ASPR of 9.90 (95% UI: 7.7 to 13.3) per 100,000, and an ASYR of 0.6 (95% UI: 0.4 to 1.0) per 100,000 in 2019, despite the decreasing trends from 1990 to 2019 (Fig. 5A, C and E). Road injuries served as the second highest ASIR of 26.3 (95% UI: 16.1 to 40.8) per 100,000, ASPR of 6.5 (95% UI: 5.3 to 8.2) per 100,000, and ASYR of 6.5 (95% UI: 5.3 to 8.2) per 100,000 in 2019, showing the increasing trends from 1990 to 2019 (Fig. 5A, C and E).

In different age groups, facial fractures due to falls remained at a high ASIR at younger ages and rapidly increased at older ages (Fig. 5B). The incidence rate of facial fractures due to road injuries peaked at ages 20–24 and then decreased with age (Fig. 5B). The ASPR and ASYR of facial fractures due to falls steadily increased with age, while the ASPR and ASYR due to road injuries peaked at older ages (Fig. 5D and F).

In terms of the main causes for the incidence, prevalence, and YLDs of facial fractures, the ASIR, ASPR, and ASYR were higher in men than in women (Supplementary Figure S1A, S1C, S1E). For the facial fractures caused by falls, the ASIR, ASPR, and ASYR were higher in young men than in young women, while those in older women over 70 years old were higher than those in older men (Supplementary Figure S1B, S1D, S1F).

## Discussion

Facial fractures can impair facial function, ruin facial aesthetics, and even cause psychosocial disorders, bringing a heavy burden to individuals, families, and nations [3, 6] In this study, we obtained the epidemiologic data on facial fractures from the GBD database, and analyzed the incidence, prevalence, and YLDs in 2019 and the temporal trends from 1990 to 2019. Fortunately, the global incidence, prevalence, and YLDs rates decreased from 1990 to 2019, reflecting the positive effects of prevention policies and social development. However, the burden of facial fractures varies considerably across countries. The burden was relatively high in countries such as New Zealand, Slovenia, and Australia, while lowest in the Democratic People’s Republic of Korea. Notably, the burden continues to increase in some countries, such as Syrian Arab Republic and Central African Republic.

From 1990 to 2019, the world population increased from 5.3 billion to 7.7 billion, meaning an increase of 45% [17]. It was found that the global incident cases of facial fractures increased from 8.9 million cases in 1990 to 10.6 million cases in 2019, corresponding to an increase of 19%. The increase in incident cases was slower than that of the global population, reflecting the effective control of facial fractures. In Central and Eastern Europe, there has been a decrease in the number of cases, possibly due to small population changes and well-controlled fractures. However, the combination of population growth and poor fracture control in East Asia, North Africa, and Middle East has led to a significant increase in incident cases.

In general, males tend to suffer a higher burden of facial fractures than females. The ASIR, ASPR, and ASYR were higher in men from 1990 to 2019, which is consistent with previous reports of outpatient cases [9, 10]. This may be due to the fact that men are exposed to more risks in their daily lives, such as road traffic accidents, interpersonal violence, intense sports, or alcohol use [11, 21–24]. Similarly, these recreational activities may be responsible for the highest incidence of facial fractures among 20–24 year olds. Additionally, the incidence of facial fractures in the elderly steadily increases with age, with a higher incidence in women after the age of 80 years. Falls are the leading cause of facial fracture in elderly patients, and the risk of falls increases with age, which may be attributed to cognitive impairment, strength deficits, sensory disabilities, medication use, or osteoarthritis [25]. Particularly, postmenopausal women were more susceptible to osteoporosis due to the loss of estrogen protection, resulting in a sharp increase in the incidence of facial fractures [26]. With the gradual aging of the world’s population, it is vital to prevent facial fractures caused by falls in the elderly [27].

Road injuries are the second cause of facial fractures, contributing to the increase in new cases yearly. Economic development has led to an explosion in the number of motor vehicles and traffic flow, increasing the occurrence of road accidents and facial fractures. The limited protection afforded to motorcyclists and cyclists during traveling is a major cause of road injuries. The use of seat belts, airbags, and helmets has been demonstrated to be an effective measure to reduce the risk of facial fractures [28, 29]. Drink driving or using a mobile phone during driving has been shown to increase the incidence of road injuries [30, 31]. Facial fractures arising from road injuries s are most prevalent among 20–24 year olds, due to the recklessness, inexperience, and high speeds of young drivers. Therefore, it is necessary to strictly enforce road regulations and improve driver safety education to reduce road injuries.

Conflict and terrorism have also exacerbated the global burden of facial fractures. We found that the Syrian Arab Republic exhibited the sharpest increase in incidence, prevalence, and YLDs rates from 1990 to 2019, which was largely attributable to regional conflicts. Similarly, as a result of the remnants of war, Afghanistan suffered the highest prevalence and YLDs rates of facial fractures in 2019. War has increased the vulnerability of people in these countries to high-energy injuries associated with facial fractures, such as shrapnel and ballistic injuries [15, 32]. However, there is a lack of medical services for patients with facial fractures in these countries, potentially leading to long-term disfigurement or disability for the victims [33]. From a global perspective, interpersonal violence is also an important cause of facial fractures, especially in the younger population.

Taking into account the impact of social and economic development, we found that the high SDI region exhibited the highest age-standardized incidence, prevalence, and YLDs rates. Typically, high SDI region has more motor vehicles, causing more traffic injuries [34]. High-energy sports, such as football, basketball, and rugby, were popular in high SDI region, leading to more sport-related facial fractures [23]. Population aging is becoming more prominent in developed countries, as better medical care would lead to increased life expectancy in high SDI region, probably elevating the incidence of fractures among the elderly. Conversely, low SDI region has difficulty in accessing adequate medical care, and many fracture cases perhaps missed. Interestingly, higher levels of treatment in developed regions reduced mortality from facial fractures, potentially resulting in an increase in the prevalence and YLDs of facial fractures.

Some measures are recommended to reduce the burden of facial fractures. Firstly, osteoporosis is a crucial cause of falls to fractures in the elderly, however, osteoporosis has been undertreated even in high-income countries [35]. It is necessary to increase public education, healthcare worker training, and medical resources on osteoporosis to relieve the disease burden caused by falls. Additionally, enforcing road regulations and improving driver safety education to reduce road injuries, such as the use of seat belts, airbags, or helmets, and the prohibition of drink driving or using mobile phones during driving. Besides, more education and attention to adolescent safety is necessary to decrease the incidence of interpersonal violence, and sport-related facial fractures. Moreover, all nations of the world need to work together to eliminate conflict and terrorism.

The present study used GBD data to analyze the global burden of facial fractures, but there are some limitations to this study. Firstly, data availability markedly affects the accuracy of results in the GBD study, and facial fractures may be underdiagnosed or underrecorded in undeveloped countries. Besides, common metrics in GBD reports include DALYs and deaths apart from YLDs. However, DALYs and deaths were not available from the online GBD database, which may be due to the fact that the burden on injuries in the GBD is calculated by the cause of injury rather than the nature of injury. Finally, more risk factors for facial fractures need to be explored, which could help explain geographic variation in the burden of facial fractures.

## Conclusions

The age-standardized incidence, prevalence, and YLDs rates decreased worldwide, however, the number of incidence, prevalence, and YLDs continued to rise due to population growth and aging. The burden of facial fractures in different countries or regions showed great variation. Falls and road injuries were the leading causes of facial fractures. This study assessed the burden of facial fractures by country, region, age, gender, SDI, and cause, which will help establish effective prevention strategies and medical resource allocation to reduce the burden of facial fractures.

### Electronic supplementary material

Below is the link to the electronic supplementary material.

Supplementary Material 1

Supplementary Material 2

Supplementary Material 3

## Data Availability

The datasets used and/or analyzed during the current study are available from corresponding author on reasonable request (https://vizhub.healthdata.org/gbd-results/).

## Abbreviations

GBD: Global Burden of Disease
YLDs: Years Lived with Disability
SDI: Sociodemographic Index
ASR: Age-standardized Rate
EAPC: Estimated Annual Percentage Change
ORIF: Open Reduction and Internal Fixation
CI: Confidence Interval
