# Supplementary figures and images for: Global, regional, and national burden of incidence, prevalence, and years lived with disability for facial fractures from 1990 to 2019: a systematic analysis for the Global Burden of Disease study 2019
Source: BMC Oral Health. 2024 Apr 10;24:435. doi: 10.1186/s12903-024-04206-9 (PMC11005257; doi:10.1186/s12903-024-04206-9)

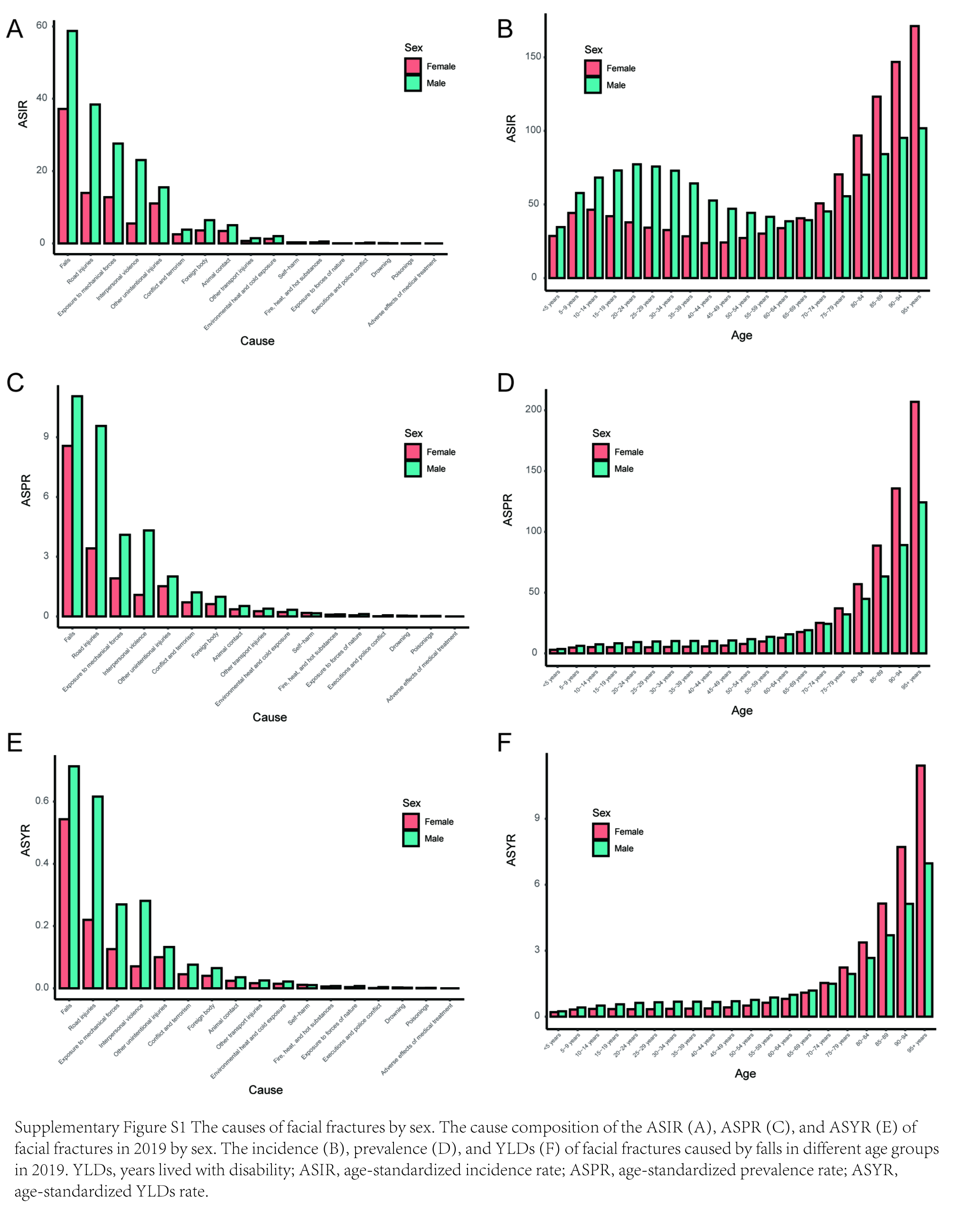

Supplement: Supplementary file 3 — Supplementary Material 3 [file 12903_2024_4206_MOESM3_ESM.tif]
